# The effect of exercising in different environments on heart rate and power output among older adults–a randomized crossover study

**DOI:** 10.1371/journal.pone.0275886

**Published:** 2022-11-02

**Authors:** Jonas Ahnesjö, Peter S. Karlsson, Patrick Bergman

**Affiliations:** 1 Department of Sport Sciences, Linnaeus University, Kalmar, Sweden; 2 Department of Economics and Statistics, Linnaeus University, Växjö, Sweden; 3 Department of Medicine and Optometry, eHealth Institute, Linnaeus University, Kalmar, Sweden; University of Innsbruck, AUSTRIA

## Abstract

**Background:**

A growing body of evidence suggests that exposure to nature is beneficial for human health. However, the observed health effect of nature may be mediated by physical activity and that humans are physically active at a higher intensity outdoors compared to when they are physical active indoors.

**Objective:**

This study examines the variation of heart rate and power output for a fixed rating of perceived exertion in a group of healthy older adults in three different environments representing three levels of exposure to nature.

**Methods:**

To this randomized, 3-by-3 crossover design study, healthy older adults (≥65 years) were recruited from local gyms. All participants participated in three experimental conditions; indoors, simulated outdoors and outdoor environments, in a randomized order. The participants exercised for 20 minutes at an intensity equivalent to a rating of 11–13 on the Borg scale for perceived exertion (RPE). Measurements of heart rate, power output (Watt) and ratings of perceived exertion were taken at minutes 1 to 6 and at minute 20. To examine the effect of the environment on heart rate and power, linear mixed models were used.

**Results:**

In all, 48 participants (56% females) were included in the analysis. No significant main effects on the outcomes were observed for power output (p = 0.073, η^2^ = 0.04) or heart rate (p = 0.067, η^2^ = 0.04)

**Conclusion:**

No significant effect on the outcomes was observed. However, borderline significant outcomes for power output or heart rate outdoors in nature, along with previous studies in the field, indicates that such an effect cannot be completely ruled out, but any effect is likely to be small. Future research examining health benefits of the independent exposure to nature are encouraged to adjust for the dose of physical activity.

**Trial registration:**

**ID:** ISRCTN22230544.

## Background

Humans have, throughout the evolutionary process, always adapted to the surrounding environment. It is therefore fair to assume, in line with the biophilia theory [1], that we experience a mismatch between evolutionary adaptations and our modern urban way of living. One of the more obvious examples of this is the obesity epidemic, a result of living in an obesogenic environment [2]. Another example is the synthetization of Vitamin-D and associated production of melanin (acting as UV-protection) is dependent upon exposure for UV-radiation, something that historically only have been possible to obtain outdoors. There is no reason to believe these examples are unique. A reasonable assumption is that human physiology and psychology are not only influenced by, but also to some degree dependent upon, the natural environment in which we have evolved. This has been described as an *added effect of nature* [3]. There is a growing body of evidence supporting the hypothesis that nature is beneficial for human health [3–7]. These positive health effects include for instance; reduced blood pressure [8], shorter time hospitalized after surgery [9] and improved global health [8, 10–12]. Merely residing close to a green space is associated with lower mortality rates independently of sociodemographic background [13]. Combined, the association and effect of nature on psychological health related outcomes such as mood and wellbeing have been extensively examined [14, 15]. Natures influence on human physiology is shown in several reviews [3–5] but this area is less understood.

Firstly, these reviews show that it is predominantly factors associated with stress such as blood pressure and cortisol levels that have been investigated limiting a broader understanding of physiological health effects of nature.

Secondly, previous studies have mostly been focused on relatively young and healthy participants [3] and information on older adults is limited.

Thirdly, most studies have not controlled for relevant confounders such as physical activity. In fact, time spent in nature might be a proxy for being physically active and that any associations observed with nature are mediated by physical activity. Physical activity is defined as any bodily movement created by skeletal muscles and that increases energy expenditure above that during rest [16]. The dose of physical activity is given by duration * frequency * intensity of physical activity and an overwhelming amount of evidence show a clear dose-response relationship between dose of physical activity and morbidity and mortality [17]. Failing to adequately control for the dose of physical activity may obscure the true relationship between nature and physiological responses. People tend to be active at a higher intensity outdoors in nature [18, 19] or rate their perceived exertion lower for a given intensity [20, 21] compared to when they are active indoors. Thus, any health benefits from nature may depend on a higher physical activity dose outdoors in nature compared to physical activity indoors.

Finally, these studies share the same limitation that the effects of the environment have been measured by altering not only the environmental setting but also the type of activity, *e*.*g*. Mieras *et al* compared actual cycling outdoors with cycling on a stationary bike indoors [19] and Hassmén compared treadmill running indoors with actual running outdoors [18]. Thus, failing to isolate the natures effect on the outcome. In addition, research have shown that being outdoors in nature may not be crucial, but rather just enjoying the scenery while being indoors could induce positive health effects [9]. Thus, investigating if it would be sufficient to simulate an outdoor environment indoors is interesting. Therefore, we examined the variation of heart rate and power output for a fixed rating of perceived effort in a group of healthy older adults by using stationary cycle ergometers in three different environments representing three levels of exposure to nature; indoors, indoors in a simulated natural environment (“simulated outdoors”) and outdoors in a natural environment. We hypothesize that when exercising outdoors the participants will work harder at a similar perceived degree of exertion resulting in an unconscious higher dose of physical activity outdoors compared to indoors.

## Methods

### Participants

**Fig 1:**
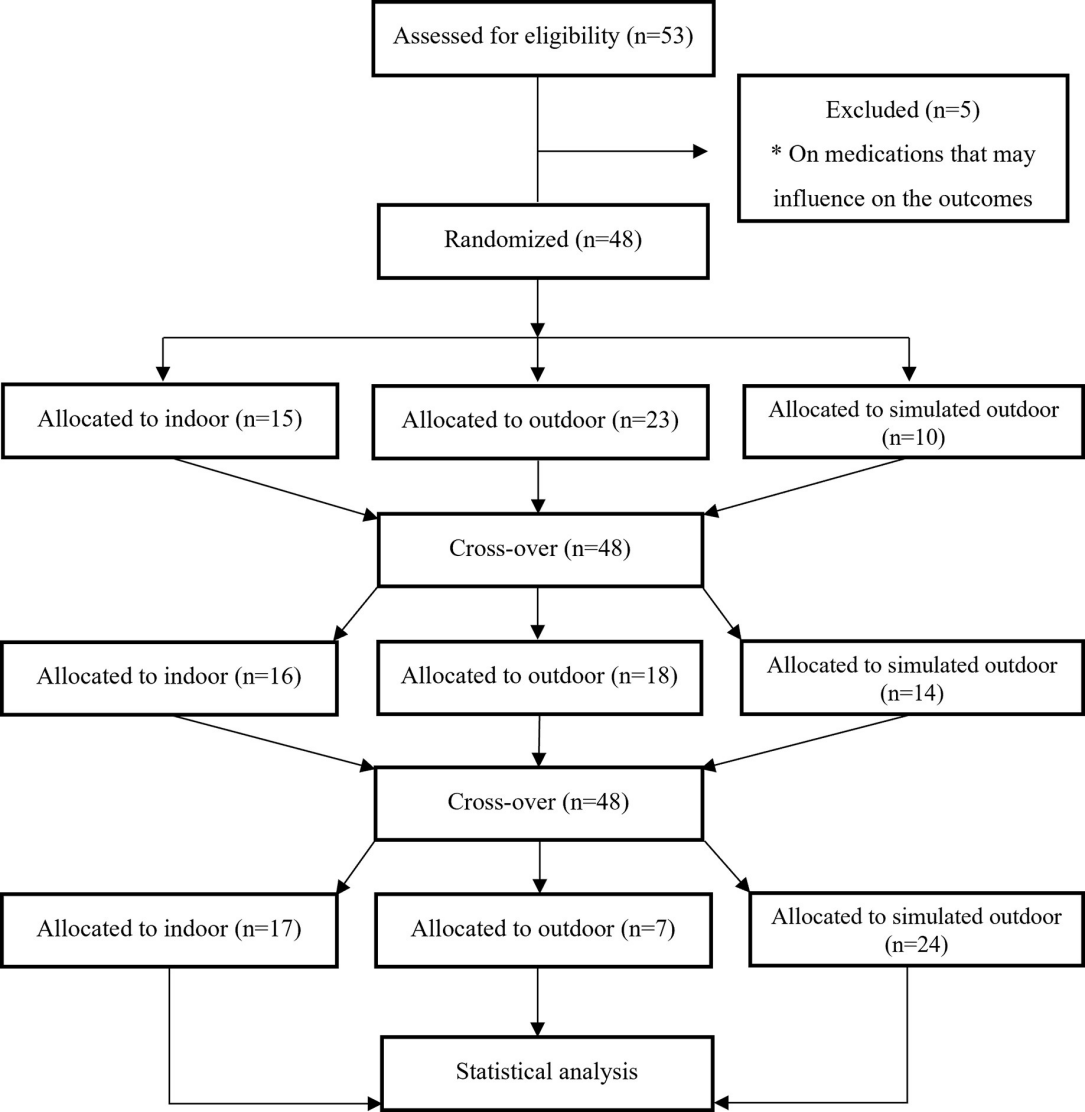
Flowchart showing the flow of participants through the trial.

According to an a priori power analysis, approximately 42 participants would be needed to detect an effect size of $\hat{f}$ = 0.2 in power output, given a α-level of 0.05 and a 1-β-level of 0.8. The calculation were made in G*Power [22]. The effect size was chosen since previous studies have shown effects of this magnitude [3]. In all, 53 healthy men and women were included in the study (see flowchart in Fig 1 for details). The participants were 65 years or older and were recruited from local gyms providing classes to older adults. To be eligible the participants had to rank their physical fitness as ≥ 6 on the Wisén scale [23], which is equivalent to being able to walk or cycle at a moderate pace for at least 30 minutes. They could not have any medical illness or be taking any medication that could have influenced any of the measurement.

### Ethical considerations

Signed informed consent was obtained from all participants. The study was approved by the Regional Ethics Review Board in Linköping (Dnr 2015/96-31). All research methods were performed in accordance with relevant guidelines and regulations, see consort checklist (S1 File) and study protocol (S2 File)

### Experimental design

**Fig 2:**
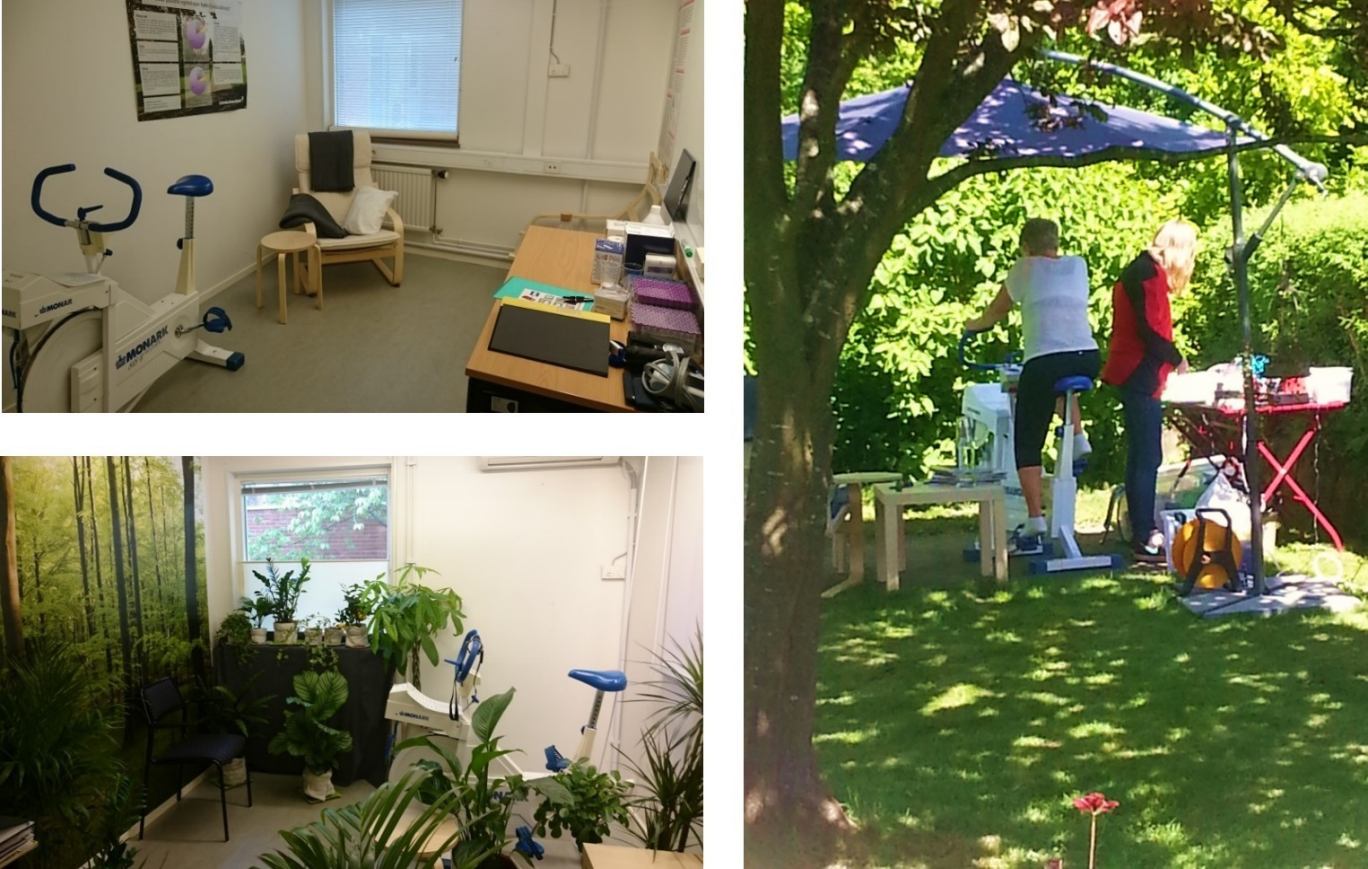
The three different experimental environments representing three levels of exposure to nature: Indoors, simulated indoors and outdoors.

All participants participated in three experimental conditions. The sequence in which each participant was exposed to the conditions was randomized. Given the three experimental conditions there are six sequences in which the participating subjects can perform the study. The specific sequence for each participant was determined using a random number generator created in R. The three experimental conditions were set up to try and represent three levels of exposure to nature, from indoors through simulated outdoors to outdoors in nature (Fig 2). The indoor condition consisted of a plain room containing only the equipment needed to perform the experiment. The simulated outdoors condition was identical to the indoor condition but decorated with a lot of green house plants, the wall the participants faced was wallpapered with a photo of a beech forest, sounds of nature were played (blackbird (*Turdus merula)* song), and daylight was simulated using four fluorescent tubes (Bio Vital, T5 EQ 28W/958). The outdoor nature condition was a lush suburban garden with the bike ergometer facing a deciduous forest. The outdoor site was selected to minimize obvious disturbing urban sounds such as traffic, which ensured the sensation of being in nature.

Even though not originally planned we subsequently included a wash out period of at least two weeks between test occasions to allow each participant to fully recover between trials. This measure resulted in a somewhat prolonged trial period than initially planned (May-June) thus the trials were conducted also during August-October. All tests were conducted in the southeast of Sweden during periods with comparable mean meteorological conditions (mean temperature ± SD = 17.0 ± 3.9°C). The outdoor trials were only performed during favorable weather conditions *i*.*e*., no precipitation and no strong wind (mean wind speed ± SD = 1.1 ± 0.4 m*s^-1^). If the weather was not favorable, we took a pragmatic approach and reallocated the subject to either the indoor or simulated indoor condition. This caused an unbalanced experiment (Fig 1).

### Procedure

All test leaders followed the same standardized protocol and prior to the study they were trained in all relevant procedures. The participants were instructed to avoid any strenuous physical activity in the 48 h before the test and to avoid eating 2 h before arriving at the test site. On arrival, the participants measured for weight and body composition using bioelectrical impedance analysis (BIA) on a Tanita BC-545 Body Composition Analyzer (Tanita, Inc., Tokyo, Japan). Height was measured on a SECA 217 stadiometer (SECA medical measurements and scales, Hamburg, Germany). They were fitted with a heart rate monitor (Polar RS800CX, Polar Electro Oy, Kempele, Finland). The resting heart rate was noted after ten minutes of sitting down quietly. Immediately after the baseline measurement was taken, the participants started the exercise on the ergometer. Based on previous research in the field where participants have exercised for 15–20 minutes [3, 20] the participants cycled for 20 minutes on a manually braked ergometer (Monark 818E, Monark Exercise AB, Vansbro, Sweden). Because the ergometer bike used was equipped with a manual brake, a fixed cadence was needed to be able to compute power output, thus, the participants was instructed to pedal with a cadence of 60 revolutions per minute. Participants pedaled at a constant moderate intensity, defined as an intensity where exertion is rated by the participant as between 11–13 on the Borg ratings of perceived exertion 6–20 scale [24]. If the rating was lower than 11, the test leader increased the resistance and if it was above the test leader decreased the resistance so that the rating of perceived exertion was within the moderate intensity. This is a deviation from the original study protocol were both heart rate and RPE was intended to guide the intensity. We did this since we wanted to examine the physiological response, e.g., heart rate to different environments and to let the subjective effort guide the resistance.

A built-in light indicator on the ergometer guided the participants to keep the correct cadence of 60 RPM (green light). We registered the heart rate, power output and RPE at minutes 1 through 6 and at minute 20 when the trial was over. These measurement points were selected so that we could estimate the participants’ aerobic capacity [25]. However, as the outcome from this test is outside the scope of this study that data is not presented. Power output (Watt) was computed according to the manufacturer’s instructions as

Work (KPM) = breaking load x distance

Assuming a KP of 2 and 1 RPM = 6 meters distance (the circumference of the flywheel) the KPM is = 2 x 60 x 6 = 720 KPM/min, from which power output is calculated as

Power output (W) = (KPM x 9.81 m/s2)/60 sec

### Outcome variables

To confirm that the participants experienced the same RPE in all environments we initially treated RPE as an outcome variable. Any differences in RPE between the environments had resulted in that we would have controlled for RPE in subsequent analyses. No such differences were observed (p = 0.644) however to be transparent we describe the analyses for RPE. Heart rate and power output were recorded for each of the 48 individuals over seven time points, in each of the three periods. To summarize the effect of different environments on heart rate (Beats Per Minute, BPM), power output (Watt) and RPE we calculated the area under the curve (AUC) for each participant’s performance in each time point during the workout using the PKcollapse command available in STATA software (version 14.0 StataCorp, College Station, TX). The AUC values were calculated based on trapezoid rule according to,

$$AUC=\sum_{i=t_{1}}^{t_{7}}\frac{1}{2}(c_{t_{i}}+c_{t_{i+1}})(t_{{i+1}_{i}}-t_{i})$$ (1)

where $c_{t_{i}}$ is either heart rate (BPM), power output (Watt) or ratings of perceived exertion (Borg) observed in time point *t_i_*. This resulted in 144 AUC values for each outcome variable.

### Statistical analysis

The 3-by-3 crossover design allows us to build a uniform within periods linear mixed model to estimate and test for a period effect, treatment effect and first order carry-over effect. The full model is an as follows [26]:

$$Y_{ijk}=\mu +\pi_{j}+\tau_{d(i,j)}+s_{ik}+\lambda_{d(i,j-1)}+\varepsilon_{ijk},$$ (2)

where *k* = individual 1,…, n_j_, *j* = period 1, 2, 3 period, *d*_*(i*,*j)*_ = treatment 1, 2, 3, *i* = sequence 1,…,6, *Y*_*ijk*_ = AUC for the outcome variable (heart rate, power output or ratings of perceived exertion) with suitable transformation if needed, μ = overall mean, πj = period effect (fixed effect), *τ*_*d*(*i,j*)_ = direct treatment effect (fixed effect), *s_ik_* = random effect associated with subject *k* on sequence *i* $(s_{ik}∼N(0,\sigma_{s}^{2}),\;\lambda_{d(i,j-1)}$ = carry over effect where $\lambda_{d(i,j-1)}=\left\{ \begin{array}{cc} 1 & if\;j=1\\ \tau_{d(i,j)} & if\;(j-1)>1 \end{array}\right.,\;\varepsilon_{ijk}$ = random error term $\left(\varepsilon_{ijk}∼N(0,\sigma^{2})\right)$ and *s_ik_* and *ε_ijk_* are mutually independent. a uniform across periods

The reduced model, i.e. model without carry-over effect, is as follows [26]:

$$Y_{ijk}=\mu +\pi_{j}+\tau_{d(i,j)}+s_{ik}+\varepsilon_{ijk}.$$ (3)

Due to technical failure, some observations are missing though the rate is very low (less than 3% overall), but to preserve the statistical power in our analyses we have chosen to impute the missing values so that we have a complete data set of AUC values for the three response variables. For the imputation analysis we entered all information available regarding heart rate or power output. Since the missing data are considered missing completely at random (MCAR), according to Rubin’s taxonomy [27], and only a very minor portion of data are missing (less than 3% overall), a single imputation method was used. The method is suitable for imputing missing data values in a repeated measurement setting [28]. This method utilizes two components, one cross-sectional (across individuals) and one longitudinal (within-individuals) [27, 28]. Imputation was made within OxMetrics software module Ox professional (Oxedit (version 7) by Jurgen Doornik, www.doornik.com), using a program written by the authors (S3 File).

In all hypothesis tests we adopt a 5% significance level to draw conclusions regarding causal relationships. Descriptive data is presented as mean ± standard deviation (SD). Any potential gender differences in baseline values were tested using independent samples t-test while differences in the baseline values between the environments was analyzed using a one-way analysis of variance (ANOVA).

The assumption of normally distributed data was tested where relevant by the Shapiro-Wilk test of normality and if necessary suitable transformation was performed. For power output, a square root transformation was performed, and for RPE, a power transformation with power equal to -1.5 was needed to satisfy the assumption of normality of the residuals in (2) and (3). Initially, we incorporated a carry-over effect of the crossover design as an extra fixed effect in the analysis, but this was dropped in the final models as it was insignificant. The analysis was performed using SAS (SAS studio version 9.4, SAS Institute Inc., SAS Campus Drive, Cary, NC 27513; USA) and the statistical package proc mixed. The residual covariance matrix chosen was “compound symmetry” [29], though “structured by periods” and “structured by treatment” were also tested. The latter two structures were rejected since the corresponding models had a poorer fit based on AICC and BIC information criteria compared to the models based on compound symmetry. Additionally, the degrees of freedom in the analysis of the crossover design are calculated according to Kenward and Roger [30, 31]. Effect sizes from multilevel models, is presented as the squared semi-partial coefficients (η^2^). The partial eta squared (η^2^) is interpreted using the rule of thumb (0.01 represents a small, 0.06 a moderate and 0.14 a large effect) [32, 33]. Figures were made using the R package of ggplot 2 [34].

## Results

In all, 48 participants (27 females) participated in this study (Table 1). No adverse events were experienced by the participants during the study. As expected, females were shorter, lighter and had a higher percentage of body fat.

**Table 1:** Descriptive data for the participating participants. P-values are from an independent samples t-test.

	Female (n = 27)			Male (n = 21)			
	Mean	±	SD	Mean	±	SD	p
Age (y)	70.4	±	3.4	70.7	±	3.4	0.795
Height (cm)	164.0	±	5.5	174.9	±	4.8	< 0.001
Weight (kg)	68.7	±	10.8	81.9	±	11.4	< 0.001
Body fat (%)	33.5	±	5.1	25.5	±	6.0	< 0.001

There was no difference in any of the dependent variables across the different environments at baseline, One-way ANOVA p>0.05 (Table 2).

**Table 2:** Mean baseline values across the three environments.

	Indoors			Simulated outdoors			Outdoors		
	Mean	±	SD	Mean	±	SD	Mean	±	SD
Heart rate (BPM)	65.5	±	12.5	64.9	±	9.4	64.7	±	9.4
Power output (W)	67.5	±	16.7	61.8	±	18.2	68.2	±	24.0
Ratings of perceived exertion (Borg)	10.7	±	1.2	10.8	±	1.3	10.6	±	1.5

The AUC for RPE were similar in all three environments (p = 0.644, η^2^ = 0.001) indicating that the subjects perceived their effort as similar in the various environments.

**Fig 3:**
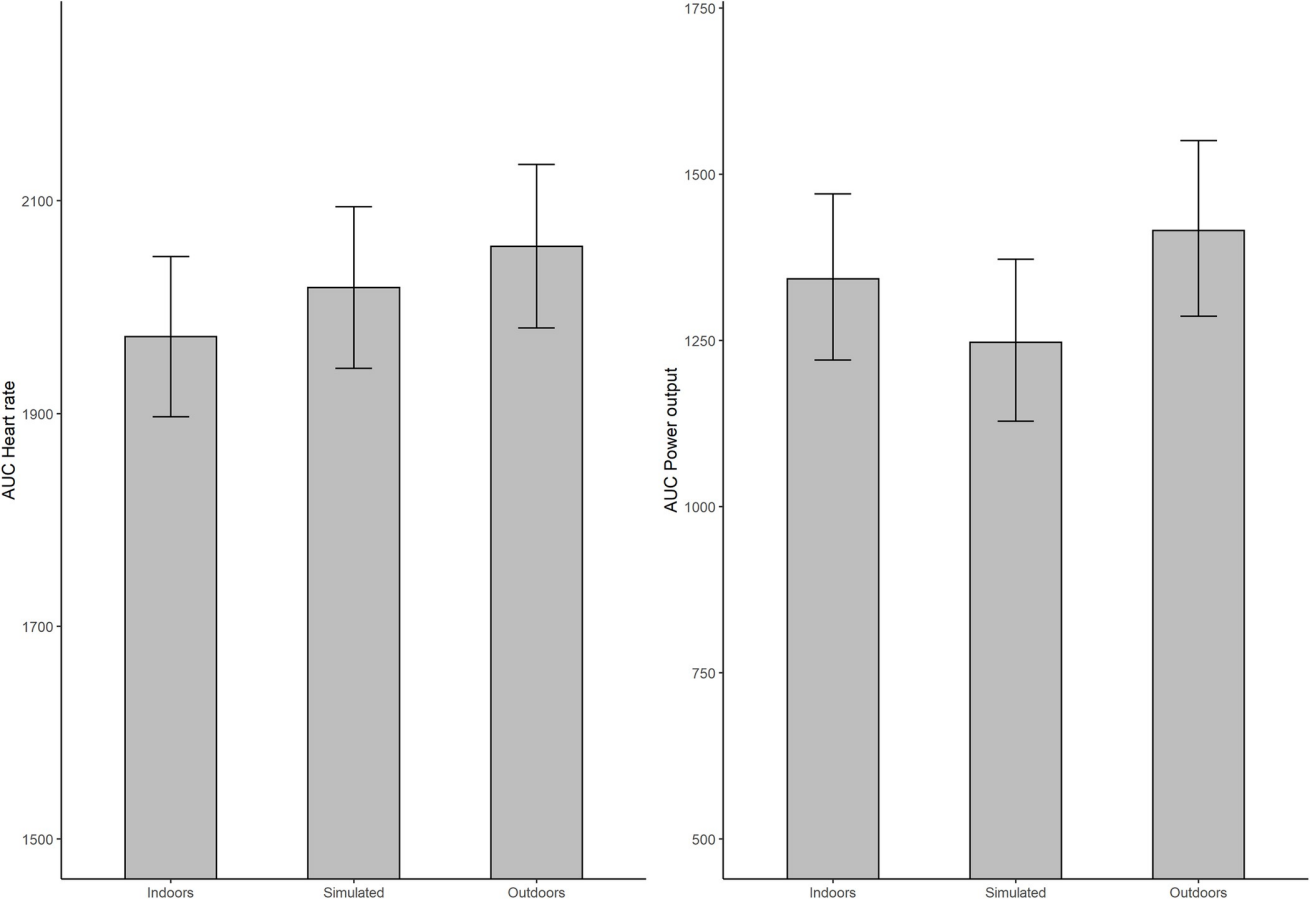
The untransformed AUC for heart rate and power output across the three environments.

A close to significant main effect of environment on the AUC for power output was seen (p = 0.073, η^2^ = 0.04), (Fig 3).

The AUC for the heart rate response was higher (albeit not significant) in the outdoor environment (p = 0.067, η^2^ = 0.04).

**Fig 4:**
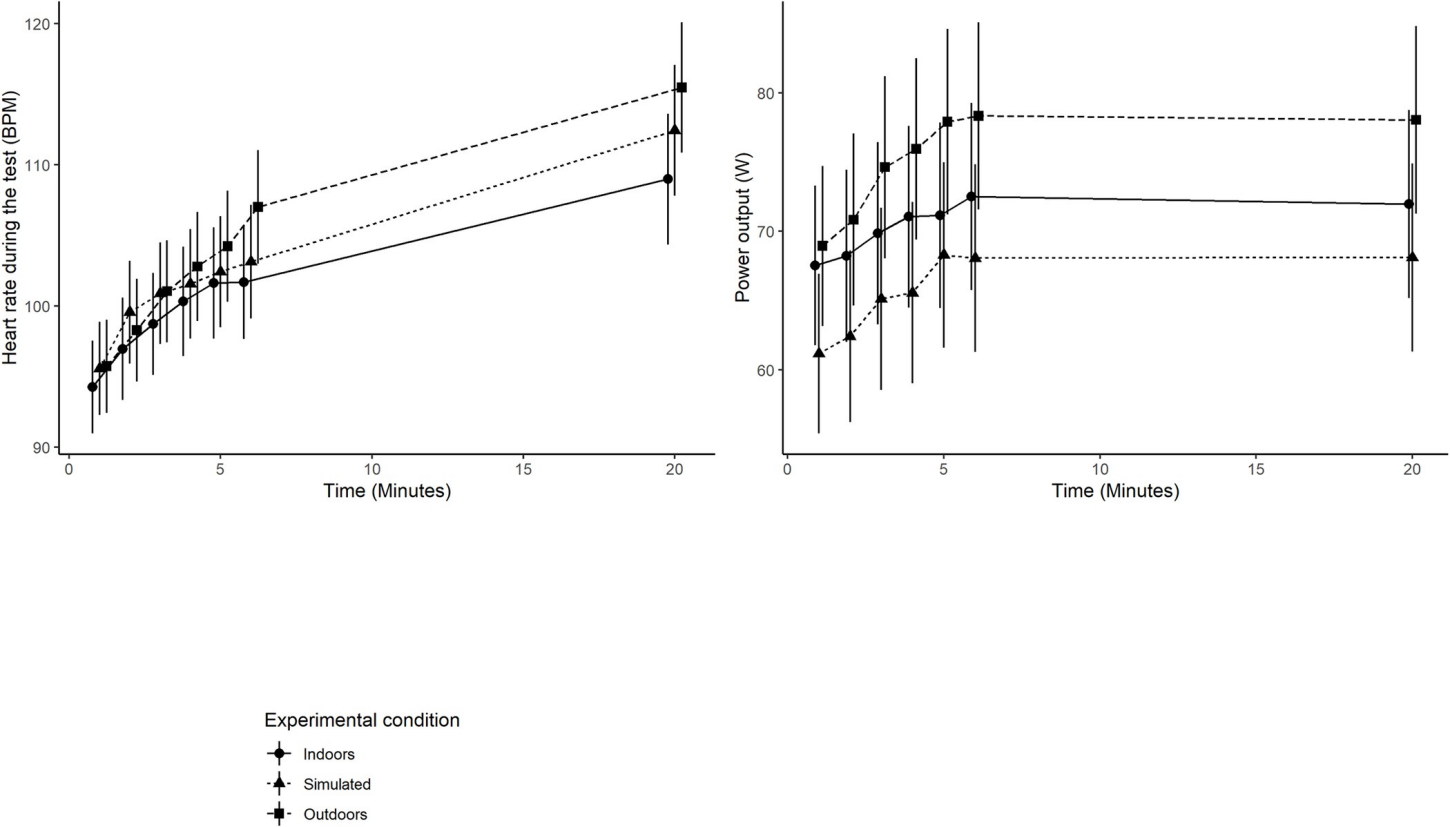
The crude mean values and the associated 95% CI for each measurement point in the different environments for heart rate and power output.

In Fig 4 the crude mean values for each measurement point in each environment for heart rate and power output are displayed.

## Discussion

In this randomized crossover trial, we observed that the AUC for heart rate and power output were numerically higher in the outdoor condition compared to the other two conditions despite identical RPE. However, the difference was not sufficiently to reach statistically significant effects in either heartrate or power output (p = 0.067 and p = 0.073 respectively). Even though, our results did not support the hypothesis that participants exercise at a higher intensity outdoors compared to indoors. The close to significant findings in this study indicates that more studies are needed to study the physiological responses to being in the nature.

The present study could not verify previously conducted studies [18–21]. However, our study is not directly comparable to the other studies in several aspects. Hassmén showed that running outdoors resulted in a substantial higher heart rate compared with running on a treadmill indoors and suggested that the ratings of perceived exertion should be adjusted so that when running on a track outdoors the rating should be lower in order to get similar response as indoors [18]. Mieras et al showed that cycling outdoors resulted in significantly higher power output compared to indoors given the same perceived effort [19]. They hypothesized that cycling outdoors is distracting and the environment draws attention from the actual effort. In contrast to our study, they did not standardize the experiment sufficiently to identify a true effect of nature given that they were on stationary ergometers while indoors but cycling on regular bikes while outdoors. In addition, they performed their experiment on trained cyclists. In a similar experiment to ours, Rogerson et al., used the same equipment indoors and outdoors but instead of letting the perceived exertion guide the intensity, their participants exercised at 50% of heart rate reserve in two environmental settings (indoors vs outdoors) [20]. In their study RPE was lower (non-significantly) outdoors compared with indoors. However, given that RPE is a relatively blunt instrument compared to heart rate, it is likely that any physiological response to exercising in nature compared to indoors may be obscured. In addition, the participants spoke more to each other during the outdoor session compared to the indoor session, which may be an indication of enjoyment. Using a slightly different approach Calogiuri and coworkers let their participants walk either in a natural environment or on a treadmill while wearing virtual reality googles showing the same route as in the natural environment [21]. They observed that even though the speed and heart rate was identical between the conditions, the respondents rated their effort higher when walking on the treadmill. Further evidence that outdoors affects behavior comes from evaluations of ordinated “exercise on prescription”. The patients increased their adherence to the treatment if they were prescribed outdoor exercise compared to indoor [35]. One of the largest studies investigating the effect of exercising in different environments involved 11,000 participants, but it is based on self-reported rather than objectively measured data [36]. That study found that those people who exercised both indoors and outdoors, and those that only exercised outdoors, were more active compared to those who only exercised indoors.

Previous findings, possibly along with ours, indicate that one of the potential reasons to why an added benefit of nature on various health related outcomes have been observed may be because it is mediated by a higher dose of physical activity outdoors in nature compared to indoors. However, the added benefit of nature is likely to be small as indicated by our relatively modest effect sizes (η^2^ = 0.04). More well-designed studies, using objective measurements and on different exercise intensities as well as different populations, are needed.

We did not find any clear evidence for a dose-response relationship regarding the gradient of exposure to nature. While exercising outdoors appeared to result in a somewhat higher intensity without an accompanying increase in the perceived exertion compared to the other two environments, power output and heart rate behaved differently in the indoor and simulated outdoor environments (Fig 3). This outcome is surprising because according to theory, power output and heart rate is strongly correlated, that is a higher workload should be accompanied with an increase in heart rate [25]. A possible explanation for this discrepancy may be that the simulated environment does not truly represent a half-way point between indoors and outdoors. The simulated outdoor environment may instead be too artificial and therefore confusing and possibly even impose psychological stress, thus increasing heart rate [37].

### Strengths & limitations

The main strength of this study is its experimental design. The randomized crossover design allows us to better model the effect of different environments without between-group variation given that the same participants are included in each arm of the experiment. Despite this, our study is not without limitations. Firstly, quantifying exposure to nature is complicated. The natural environment consists of an almost infinite number of possible combinations of environmental parameters including temperature, humidity, wind, sunlight etc as well as scenery. Indoors on the other hand is compared to outdoors almost to be considered as constant with almost no fluctuations in temperature or humidity for example. Even if we tried to create a gradient from indoors to outdoors via a room simulating outdoors it is difficult to be sure we achieved this. Instead, we saw that the simulating the outdoor environment might have caused confusing effects. Secondly, our study was comprised of a non-random sample of active older adults limiting the ability to generalize our findings to a wider population. Thirdly, the exercise in our experiment was performed at quite a low intensity and it is possible that the effects are greater and significant if this experiment was repeated but with higher intensities of exercise.

In summary, our results along with previous research, may indicate that exercising outdoors may be beneficial for health among older adults through the increased dose of physical activity. This is an important finding since the proportion of older adults is increasing around the world. Estimates from Statistics Sweden (www.scb.se) suggest that in the year 2050, people over 60 years will double in numbers. This demographic transition is a challenge for societies across the world, namely, how to keep this age group as healthy as possible. Outdoor recreation in general and physical activity in particular are important aspects in achieving this.

## Conclusions

Even though no significant main effects of nature were found in the present study the low p-values makes it difficult to rule out that such an effect is present. Taken together it appears as if, at least in part, the suggested positive effects of exposure to nature on health outcomes may be mediated via the well-known health benefits of physical activity. Therefore, we encourage others to control for the dose of physical activity in future studies examining the putative health effects of nature.

## Supporting information

S1 File Consort checklist. (DOC)

S2 File Study protocol. (DOCX)

S3 File Technical annex. (DOCX)
