# Supplementary material for: The effect of exercising in different environments on heart rate and power output among older adults–a randomized crossover study
Source: PLoS One. 2022 Nov 2;17(11):e0275886. doi: 10.1371/journal.pone.0275886 (PMC9629597; doi:10.1371/journal.pone.0275886)
Supplement: S2 File — (DOCX) [file pone.0275886.s002.docx]

**HEALTH EFFECTS OF RECREATION OUTOORS**

BACKGROUND

In Sweden, > 90 % of the adult population claim that being outdoors in nature gives some meaning to their life (Fredman et al, 2008). Kaplan och Kaplan (1989) have shown that nature is a place to visit in order to achieve restorative effects. Thus, recreation outdoors may be an important aspect of human’s everyday life. Common belief also tells us that being outdoors is good for the individual health. The positive health effects of nature have been observed in a number of studies (*e.g.* Velarde, G *et al.* 2007). These positive health effects includes for instance; reduced blood pressure (Hartig, Evans *et al.* 2003), shorter time hospitalized after surgery (Ulrich 1984) and improved global health (Hartig, Evans *et al.* 2003; Hansmann, Hug *et al.* 2007; Tenngart Ivarsson and Hagerhall 2008; Korpela, Ylen *et al.* 2010). Merely reside close to a green area reduces morbidity and mortality independent of socioeconomic status (Michel & Popham, 2008).

But most of these studies are based on subjective measurements of health, although studies using objective measures exist (Hartig, Evans *et al.* 2003; Berman, Jonides *et al.* 2008). Most studies also rely on observational study designs which hamper the ability to draw any conclusions regarding causal effects.

In this study we experimentally investigate if the positive effects of nature are reflected also in physiological parameters in a group of healthy elderly subjects (65 years or older). Our overall research hypothesis is that the nature itself has a positive direct influence on health and not only as an effect of physical activity in a natural environment. More specifically we will investigate; the effect of nature on markers of sympathetic tonus, inflammatory and immunological response, recovery after exercise, vitamin D and on brain health, controlled for potential confounding factors such as body composition, level of physical activity, time spent outdoors, socio-demography and other factors believed to be associated with either exposure to nature or the physiological parameters.

METHODS

**Subjects**

Healthy men and women aged 65 or older will be recruited from local sport clubs and gyms. Before entry to the study the subjects will conduct a health declaration in which answer questions regarding their health. They will also rate their aerobic capacity in the Wisen RPC-scale (Wisen AG, Farazdaghi RG & Wohlfart B, 2002). To be eligible the subjects should rate six or higher on the scale. Subjects with a diagnosis or on a medication that may influence on the measurement will be excluded, such as subjects with a peacemaker, chronic inflammatory diseases or uncontrolled hypertension. A power analysis shows that in order to have a 90 % power to detect a moderate effect (effect size = 0.4) between factors, given an alpha level of 0.05, in a repeated measures ANOVA, 51 subjects are needed. To cover for losses due to drop out we target to recruit 60 subjects (50 % women).

**Study design**

This is a cross over study in which three experimental conditions will be tested; indoors, a simulated outdoor environment and outdoors in a natural outdoor environment. The indoor environment will consist of a single room just decorated with what is needed for performing the experiment, such as measurement equipment, a bike ergometer and so on. The simulated outdoor environment will be in a room identical to the indoor situation but decorated with green plants, daylight lamps and in all other aspects contain as much of outdoor features as possible. The last condition is outdoors. We will select a site in a forest near a city, but not close to any major roads or other things that can interfere with the sensation of being in nature.

All subjects will participate in each experimental condition and given that there are three experimental conditions there are six different orders (A-F) that the participating subjects can perform the experiment (table 1). The exact order each subject will conduct the experiments in will be randomized.

Measurements will take place during spring and early summer (April-June) in order to avoid too cold or too hot weather and to obtain comparable conditions regarding climatological parameters indoors and outdoors. To be able to control for the different climate conditions we will also measure temperature, humidity and wind speed at each location and test trial.

**Table 1** Given the three experimental conditions there are six orders in which the participating subjects can perform the study.

|  | Indoors | Simulated outdoors | Outdoors |
| --- | --- | --- | --- |
| A | 1 | 2 | 3 |
| B | 1 | 3 | 2 |
| C | 2 | 1 | 3 |
| D | 2 | 3 | 1 |
| E | 3 | 1 | 2 |
| F | 3 | 2 | 1 |

**Procedure**

The subjects will be instructed to avoid any strenuous physical activity 48 h before the testing. They will also be instructed to avoid eating 2 hours before they come to the test site.

When the subjects arrive to the test site, independent of experimental situation, they will be fitted with the heart rate monitor, and a venous catheter is inserted in the antecubital vein. After those initial procedures the subjects will sit down and rest quietly. After ten minutes the resting heartrate and blood pressure will be measured according to previous description and the baseline blood sample is taken. After that the subjects will start the exercise on the bike ergometer. The ergometer will be adjusted according to the height of the subject and the initial resistance will be determined according to gender and training background.. As a rule of thumb the women starts at 50-75 Watt and the males at around 100 Watt. During the first six minutes of the trial the subjects will perform a submaximal test for aerobic capacity (Åstrand-test). During these six minutes, heart rate and resistance will be adjusted inorder to reach steady state with a heart rate of around 130 and a rating of 11-13 at the Borg RPE scale after six minutes. Based on the heart rate the subject’s maximal aerobic capacity can be estimated (Åstrand & Ryhming 1954). The subjects will however continue to bike for another 14 minutes before the test is terminated. Every third minute heart rate, Borg rating and the resistance (W) will be recorded with the aim of keeping the heart rate steady during the entire session. After the termination of the physical exercise the subject will be seated to rest quietly for 120 minutes during which a series of measurements will be conducted. At minute 10, 20, and 30 after terminated physical exercise, the heart rate, Borg rating and blood pressure will be measured and small amounts of blood will be collected for the on-site analyses (Lactate, glucose and count of white blood cells). At baseline, immediately after the end of the physical exercise, 30 and 120 minutes post exercise larger blood samples will be collected for later analysis of biomarkers related to the research questions. These samples will be transported to the department of clinical chemistry at the hospital for preparation and storing at -80°C within 4 hours after the baseline measurement was taken.

**Measurements**

The main interests of this study is the physiological effects of nature on human health. More specifically, we are interested in effects on the sympathetic nervous system (Blood pressure, heart rate variability and biomarkers (cortisol)), on immunology (white blood cell count and biomarkers (C3 and C4) inflammation (TNF-a, IL-6), recovery after exercise (Heart rate, lactate, glucose and insuline). But we will also examine several potential confounding variables, such as level of physical activity and body composition.

Blood pressure

Blood pressure will be measured using a hand held automatic blood pressure meter (WelchAllyn ProBP 3400, WelchAllyn Inc, Skaneatels Falls, USA). In order to achieve best possible estimate of resting blood pressure the subjects will be seated for a minimum of 10 minutes prior the first measurement. A second measurement will be taken three minutes after the first and if those two measurements deviates by more than 5 % a third measure will be taken. The average between the two closest measurements will then be used as an indicator of resting blood pressure. At all subsequent measures only one measurement will be taken.

Heart rate and heart rate variability

The heart rate as well as the heart rate variability will be measured using a heart rate monitor (Polar RS800CX, Polar OY, Kempele, Finland). The heart rate monitor is capable of detecting the R-R intervals and store the data as two variables, the average heart rate during the last five seconds and the time between two R-R intervals in milliseconds.

Physical activity

Physical activity will be assessed by accelerometry. Accelerometers are small lightweight devices that detect bodily movements in three planes. The particular devices used in the study Actigraph GT3X (ActiGraph LLC, Pensacola, FL, USA) are also capable of measuring illuminance (Lux), making it possible to measure the time subjects are outdoors compared to indoors and also the amount of physical activity that takes place indoors or outdoors respectively. The accelerometers are fitted snuggly around the waist at the right hip using an elastic waistband. It’s worn all time awake except during water based activities such as showers. This means that the subjects puts it on in the morning and takes it off when the go to sleep. The subjects will wear the accelerometer during the time between two test occasions (i.e. two weeks). The accelerometers do not interfere with the participant’s everyday life.

Measurement of body composition

Height will be measured using a portable stadiometer (Seca 217, Seca gmbh & co, Hamburg Germany). Body weight and body fat percentage will be measured using a bioelectrical impedance scale (Tanita BC-545, Tanita Corp, Japan). From weight and height the body mass index (kg/m^2^) will be calculated as the weight (kg) divided by the height squared (m^2^).

Borg RPE 6-20

The Borg RPE scale is a method to self-report perceived exertion (Ratings of Perceived Exertion). The scale starts at 6 which indicate no effort and goes to 20 indicating worst possible exertion (Appendix X). Moderate effort corresponds to a rating of approximately 11-13 on the scale. During the time which the subjects are riding the ergometer bikes, they will on regular basis rate their exertion so that the resistance may be adjusted such that perceived exertion remains between 11-13 according to the Borg RPE scale.

Questionnaire

Each subject will fill out a questionnaire in 4 parts (appendix X). The first part collects data about socio-demographic information, the second about physical activity, the third about the local neighborhood environment and the last about attitudes to environment and environmental preservations. Each subject will also fill out a health declaration (appendix X) in which we are interested in information related to the subjects ability to perform the study and regarding medications that may influence on the outcomes (such as beta blockers).

Blood samples

In this study we will analyze blood samples taken at regular intervals (fig 1). The blood will be taken from a peripheral venous catheter (PVC) inserted in the antecubital vein in an EDTA test tube. The amount of blood collected at each occasion will vary depending on the kind of analyses that will be performed on the specific sample. The on-site analyses do not require any large amounts of blood thus only a small (3 mL) sample will be drawn from the PVC for each on site analysis. On site we will analyse blood lactate (lactate pro2) and blood glucose (HemoCue Glucose 201 RT Analyzer). We will also count five different types of white blood cells, Neutrophils, lymphocytes, monocytes, basinophils and eosinophils and get the total number of white blood cells. The blood cell count will be made using a HemoCue WBC Diff (HemoCue AB Ängelholm, Sweden). After the on-site analyses have been conducted the remaining blood will be destroyed. At the end of each test occasion a subject will have donated approximately 80 ml blood of which 69 ml is for research purposes and the rest is due to the need for taking some blood in relation to rinsing of the catheter. In order to allow each subject to form new blood there will be at least two weeks between two test occasions. In total each subject will have donated around 240 ml blood at the end of the experiment, which corresponds to some 50 % of a normal blood donation.

**Figure 1** A timeline describing when the blood will be collected, and the amount at each occation. At each occation (T_0_-T_6_) registration of heart rate and measurement of blood pressure, blood glucose, blood lactate and count of white blood cells will be conducted.

Within four hours of the first blood sample is taken, all blood samples collected for laboratory analyses will be transported to the department for clinical chemistry at the hospital where it will be centrifuged at 2500 x G for 20 minutes and transferred to cryo tubes and stored at -80°C until the end of the data collection period. Samples will then be analysed using a a set of multiplex luminex assays. We will analyse the plasma for markers of immunology (C3, C4) inflammatory markers (IL-6, TNF-a) hormones (cortisol and Insuline), brain health (BDNF) and vitamin D.

Next generation sequencing

From the blood collected at baseline and 120 post exercise we will sort and stabilize mRNA from white blood cells using commercially available kits with the aim of performing next generation sequencing (NGS) on the mRNA of the cells to quantify the genetic response of exposure to the different experimental conditions. On a random sub-sample this analysis will be conducted at the end of the data collection period. The remaining data will be stored and analyzed at a later occasion.

TIME LINE

The measurements are planned to be conducted during spring (april-june) in order to avoid too cool or too hot temperatures. The analysis of the blood samples is planned to be conducted during early autumn after which analyses interpretation of the data will be conducted.

ETHICAL CONSIDERATIONS

Volunteers to participate in the study will be recruited at gyms and sport facilities with special activities for seniors. This procedure should help us to avoid recruiting participants that are not sufficiently fit to conduct in the study, such as subjects with underlying medical problems. The level of the exercise that the subjects will perform is comparable to a warm up or a 20 minutes brisk walk which also indicates that the overall risk for medical complications is low. This is not a guarantee that nothing may occur which is why all our field workers are trained in cardiopulmonary resuscitation. During the test indoors a defibrillator is located in the same building.

The participants may at any time without reason leave the study and withdraw all information that has already been collected about them. None of the measurements in this study can be expected to cause any considerable pain or discomfort and we do not ask any questions that may be considered questionable from an ethical wievpoint.

We cannot foresee how the usage of the outcomes from this study can be used to have a negative influence on the studied group.

IMPORTANCE OF THE STUDY

The present study can contribute to the overall understanding of the effect nature has on human health as well as performance and recovery after exercise. The study should play an important role when it comes to the identification of acute physiological effects induced by nature and the results may contribute to the promotion of being and exercising in natural environment and the usage of outdoor sport facilities. As an extension, the outcome of this project may also be used when planning and designing indoor sport facilities as well as nursery homes. It is possible that the health care system can make use of the result by for example schedule time outdoors for patients in nursery homes.

REFERENCES

Berman, M. G., J. Jonides, et al. (2008). "The Cognitive Benefits of Interacting With Nature."

Psychological Science **19**(12): 1207-1212.

Fredman, R., Karlsson, S-E, Romild, U. och Sandell, K (red.) (2008). *”Vara i naturen - varför eller varför inte? Delresultat från en nationell enkät om friluftsliv och naturturism i Sverige”*. Forskningsprogrammet Friluftsliv i Förändring: Östersund, Sweden, 2008. *[In Swedish]*

Hansmann, R., S. M. Hug, et al. (2007). "Restoration and stress relief through physical

activities in forests and parks." Urban forestry & Urban greening **6**: 213-225.

Hartig, T., G. W. Evans, et al. (2003). "Tracking restoration in natural and urban field

settings." Journal of Environmental Psychology **23**(2): 109-123.

Mitchell, R & Popham, F (2008). “Effect of exposure to natural environment on health inequalities: an observational population study”, Lancet, vol. 372(9650), pp. 1655- 1660.

Kaplan, R. & Kaplan, S. (1989). *“The experience of nature – a psychological perspective”*. Cambridge: Cambridge University Press.

Korpela, K. M., M. Ylen, et al. (2010). "Favorite green, waterside and urban environments,

restorative experiences and perceived health in Finland." Health Promotion

International **25**(2): 200-209.

Ord, K., Mitchell, R. and Pearce, J. (2013) "Is level of neighbourhood green space associated

with physical activity in green space?" International Journal of Behavioral Nutrition

and Physical Activity 10:127

Tenngart Ivarsson, C. and C. M. Hagerhall (2008). "The percieved restorativeness of gardens

- Assessing the resotativeness of a mixed built and natural scene type." Urban forestry

& Urban greening **7**: 107-118.

Ulrich, R. S. (1984). "View through a Window May Influence Recovery from Surgery."

Science **224**(4647): 420-421.

Velarde, M. D., F. G, et al. (2007). "Health effects of viewing landscapes - Landscape types in environmental psychology." Urban forestry & Urban greening 6: 199-212.

Wisen AG, Farazdaghi RG, Wohlfart B. ”A novel rating scale to predict maximal exercise capacity”. Eur J Appl Physiol 2002;87:350-7.

Åstrand PO & Ryhming I (1954) A Nomogram for Calculation of Aerobic Capacity (Physical Fitness) From Pulse Rate During Submaximal Work Journal of Applied Physiology 7 (2) 218-221
