# Supplementary material for: The effect of exercising in different environments on heart rate and power output among older adults–a randomized crossover study
Source: PLoS One. 2022 Nov 2;17(11):e0275886. doi: 10.1371/journal.pone.0275886 (PMC9629597; doi:10.1371/journal.pone.0275886)
Supplement: S3 File — (DOCX) [file pone.0275886.s003.docx]

**Appendix A: Missing data analysis**

//--------- OX Program for imputing in paneldata ---------;

#include <oxstd.oxh>

#include <oxprob.oxh>

#import <maximize>

#import <simulator>

#include <oxfloat.oxh>

main()

{

print ("\ntime=", time(), " Datum=", date(), "\n");

decl time, tom, Xt, Xtnoll, X, i, j, Delta, pt, nt, Xtmean, ettnt;

decl ettpt, columnN, D, rowmeanD, Ximpute, Impute;

tom=<>; time=timer();

// read in datafile for a specific variable observed under a one specific period ---;

// the data set is orginized as follows: rows=time points , columns=individuals ---;

X=loadmat("7.1 missing data period 1 wide format for new method Heart.xlsx"); //--- specifying file name of the datafile --;

print ("Transposed Data set with missing observations rows= individuals & columns= time points ",X');

print("", tom);

//-----------------------------------------------------------------------------------------------------------;

Xt=X'; Xtnoll=Xt; nt=rows(Xt); pt=columns(Xt);

ettnt=ones(nt,1); ettpt=ones(pt,1); Delta=zeros(nt,pt); Impute=zeros(nt,pt);

for (j=0; j<pt; ++j) {

for (i=0; i<nt; ++i) {

if (Xt[i][j] > 0.0) Delta[i][j] =1;

}}

for (j=0; j<pt; ++j) {

for (i=0; i<nt; ++i) {

if (Xt[i][j] >0) Xtnoll[i][j]=Xt[i][j];

else Xtnoll[i][j]=0;

}}

columnN=ettnt'*Delta;

Xtmean=((ettnt'*Xtnoll).*(columnN.^-1))';

D=(Xtnoll-ettnt*Xtmean').*Delta;

rowmeanD=(D*ettpt).*((Delta*ettpt).^-1);

Ximpute= ettnt*Xtmean' + rowmeanD*ettpt';

for (j=0; j<pt; ++j) {

for (i=0; i<nt; ++i) {

if (Delta[i][j] == 0) Impute[i][j]=Ximpute[i][j];

}}

print(" Number of individuals = ", nt);

print(" Number of time points = ", pt);

print("", tom);

print(" Matrix containing values to be imputed, rows= individuals & columns= time points", Impute);

}
